# A serological survey of *Toxoplasma gondii* in polish pigs from organic farms, other housing systems and in pigs of different age groups

**DOI:** 10.1186/s13028-022-00623-4

**Published:** 2022-02-07

**Authors:** Martyna Puchalska, Jan Wiśniewski, Daniel Klich, Elżbieta Gołąb, Dawid Jańczak, Justyna Sokołowska, Kaja Urbańska, Krzysztof Anusz

**Affiliations:** 1 Department of Food Hygiene and Public Health Protection, Institute of Veterinary Medicine, Warsaw University of Life Sciences-SGGW, Nowoursynowska 159, 02-776 Warsaw, Poland; 2 Department of Animal Genetics and Conservation, Institute of Animal Science, Warsaw University of Life Sciences-SGGW, Ciszewskiego 8, 02-786 Warsaw, Poland; 3 Department of Parasitology and Vector-Borne Diseases, National Institute of Public Health-National Institute of Hygiene, Chocimska 24, 00-791 Warszawa, Poland; 4 Department of Morphological Sciences, Institute of Veterinary Medicine, Warsaw University of Life Sciences-SGGW, Nowoursynowska 159, 02-776 Warsaw, Poland

**Keywords:** ELISA, Seroprevalence, Toxoplasmosis

## Abstract

**Background:**

The consumption of raw or undercooked meat, especially pork, and offal containing infective tissue cysts is suspected to be a significant route of infection with *Toxoplasma gondii*. Although the use of “animal-friendly pig production systems” ensuring direct contact with the natural environment offers ethical benefits, it limits the ability to ensure animal health; it may also increase the probability of infections by pathogens such as *T. gondii*, and thus their entry into the food chain. This study determines the seroprevalence of *T. gondii* in pigs from different housing systems and farms with different hygiene standards in Poland, as well as among pigs of different age groups from farms with high hygiene standards. In total 760 pig serum samples were examined for the presence of specific antibodies using the PrioCHECK^®^ Toxoplasma Ab porcine commercial ELISA test (Prionics, Switzerland).

**Results:**

Test results with PP ≥ 20% were regarded as positive, as indicated by the manufacturer. Antibodies to *T. gondii* were found in 193 of 760 (25.4%) tested sera. Regarding different housing systems, antibodies were found in 117 pigs: of these, 52.6% (61/116) were from organic farms, 40.9% (47/115) from farms with low hygiene standards, 5.4% (9/167) from farms with high hygiene standards and 0% (0/40) from a farm with a high level of biosecurity. Regarding age groups, antibodies were found in 76 animals on farms with high hygiene standards: 11.1% (7/63) were pigs younger than 3 months, 0% (0/60) aged 3–4 months, 12.3% (7/57) aged 5–6 months (final fattening stage) and 43.7% (62/142) were sows aged 9 months and older.

**Conclusions:**

Antibodies to *T. gondii* were most often found in pigs from organic and low-hygiene farms, as well as in pigs aged 9 months and older. Meat derived from seropositive animals can pose a potential source of infection for humans. As maternal antibodies to *T. gondii* can be present in the blood of piglets aged up to 3–4 months, serological examination is unjustified in piglets up to this age.

## Background

Recent years have seen a growth in consumer interest in animal products obtained using “animal-friendly pig production systems”, i.e., those providing contact with the natural environment; for example, pork from organic and free-range pigs [1]. Although such systems offer ethical benefits, they limit the potential for controlling animal health [2]. Their use may also increase the number of infections with pathogens, such as *Toxoplasma gondii*, and thus the possibility of them entering the food chain [3].

The life cycle of *T. gondii* is an indirect one consisting of asexual reproduction, in an intermediate host, and sexual reproduction, in the intestine of the definitive host. The parasite can use a wide range of mammals and birds as intermediate hosts, including humans and domestic pigs [4]; however, the definitive host must be a member of the *Felidae* family [5]. After primary infection, the definitive host excretes millions of oocysts with their feces [6]. After sporulation, the oocysts can infect a wide range of hosts through contaminated soil, water and feed [7], and can persist for a long time in the environment [8]. In the intermediate host, infection results in the formation of tissue cysts containing bradyzoites, which represent the terminal life-cycle stage in this host. Consumption of tissue cysts usually leads to infection in non-immune individuals [9].

Humans can become infected via three main routes: congenitally from the mother, by ingesting sporulated oocysts in soil, water and vegetables contaminated by cat feces, or by consuming tissue cysts present in the meat of infected animals [10]. Of these three, the most significant route in human infection is believed to be the consumption of raw or undercooked meat and offal containing infectious tissue cysts [11]. In addition, the prevalence of *T. gondii* infection in humans is believed to be influenced by age, geographical location, nutritional habits and hygiene standards [12]. Even so, approximately 1/3 of humanity is believed to be infected [13], with the seropositive results varying from 5 to 90% depending on region [14]. In Poland, this seropositivity ranges from 36% in the Małopolskie Voivodeship to 62.5% in the Pomorskie Voivodeship [14].

Although infection is usually asymptomatic [15], immunocompromised individuals can be at risk of serious complications, including encephalitis, brain abscesses and death [16, 17]. Pregnant women may suffer preeclampsia and miscarriage, and mental disorders and malformations can occur in the newborn child [18–20]. Even healthy humans and animals can demonstrate behavioral changes, neuropsychiatric disorders and infertility as a result of infection [21–25].

The European Food Safety Authority (EFSA) recommends that meat and meat products from pigs, especially those from organic farms, should be prioritized for monitoring, as well as meat from sheep and goats from free-range systems [26]. Among these species, pigs (pork) are considered to be one of the main sources of meat-borne infection [27]. As it is not possible to detect *T. gondii* tissue cysts during routine post-mortem examination [28], the EFSA recommends the use of serological tests to detect specific antibodies when testing pigs [29], as this can indicate presence of tissue cysts in muscles [12, 28, 30].

In Poland, the main factor increasing the probability of *T. gondii* infection is believed to be the consumption of raw pork [31]. Pork is the most widely-consumed meat in the country: in 2019, consumption amounted to 40.3 kg per capita [32]. Similarly, infection has also been attributed to tasting raw meat during meal preparation [31] and the consumption of frozen, minced pork [33].

Heat treatment is the most effective method of inactivation of tissue cysts. While heating to 67 ℃ or higher is considered sufficient to immediately kill tissue cysts, at lower temperatures, tissue cyst survival depends on the duration of heating. At 60 ℃, tissue cysts remain viable for about four minutes and at 50 ℃, for about 10 min. Most tissue cysts are also killed at temperatures of − 12 ℃ or lower but some tissue cysts may survive deep-freezing. Tissue cysts can also be killed by curing; however, survival time varies with the concentration of the salt solution and temperature [26]: one study found cysts to be killed by 3% table salt solution [26], while another found cysts to be killed by a 1.3% solution [34].

Tissue cysts are typically unequally distributed throughout an infected animal. In pigs, the highest concentrations are found in brain tissue; however, this is only occasionally consumed by humans. While the numbers of tissue cysts in muscle tissue are comparatively low, pork should still be considered as a potential source of infection for humans [35]. A particular challenge is posed by individual meat products consisting of a mixture of meat and offal derived from multiple animals [36]; in such cases, the meat and offal of a single pig may be divided between more than 600 servings of infected meat [37], and the meat from one infected pig can contaminate the entire batch of a final meat product [38].

Pigs are most often infected by exposure to feed, water and environments contaminated by oocysts [39], or by the consumption of tissue cysts present in the tissues of intermediate hosts, especially small rodents or birds [28]. Cases of intrauterine infections have also been reported [40]. Older animals tend to have a greater probability of infection [41], and this has been attributed to their increased likelihood of encountering the pathogen [15]. In addition, sows are at up to 41-times greater risk of *T. gondii* infection than finishing pigs [42].

Pig breeding in Poland is highly fragmented between smallholdings [43]. As small farms are more likely to have poor hygiene conditions and the animals are more likely to be exposed to infective forms of the parasite present in the soil, water and feed, this arrangement also promotes *T. gondii* infection [44, 45].

The aim of the present study was to determine the seroprevalence of *T. gondii* between pigs from different housing systems and farms with different hygiene standards. It also examined the presence of specific antibodies in pigs of different age groups from farms with high hygiene standards.

## Methods

### Farms

A number of farms were selected for the study *by convenience*, i.e. the farms were either located in areas subjected to the Poviat Veterinary Inspectorates cooperating with us, or had sent fattening pigs for slaughter to slaughterhouses that were cooperating with us for the study. These were observed and classified in terms of housing system and hygiene standards based on the following four documents: the SPIWET (Coordinated Program of the Veterinary Inspection) checklists, Annex IV to Regulation No. 2015/1375 of 10 August 2015 laying down specific rules on official controls for *Trichinella* in meat [46], Council Regulation (EC) No. 834/2007 of 28 June 2007 on organic production and labeling of organic products [47] and the 25 June 2009 Act on organic farming [48]. Four types of farms were distinguished in the present study: those with high hygiene standards, those with low hygiene standards, those with a high level of biosecurity, and organic farms.

The study included three farms with high hygiene standards, all of which were located in the Lubelskie voivodeship. In these farms, the herd size exceeded 200 animals. The pigs had no access to outside pens and contact with the environment was limited. The livestock buildings were protected from rodents, birds, insects and companion animals, including cats. All farms implemented rodent and insect control programs and ensured that the nutritional and microbiological quality of feed stored in closed silos was appropriate. Outside access to the livestock buildings was restricted and each visit was recorded.

The study included 13 farms with low hygiene standards, all of which were located in the Mazowieckie voivodeship. On these farms, the herd size did not exceed 50 animals. The pigs had no access to outside pens, but cats had access to the premises, and in many places, traces of rodents and insects were visible. In buildings where feed was stored and in rooms intended for animals, windows or doors were often left open and not always secured with a safety net, or the safety net was damaged.

The study also included one farm with a high level of biosecurity, and this was located in the Wielkopolskie voivodeship. On these farms, hygiene conditions were similar to those required on farms providing controlled conditions in accordance with Annex IV, Regulation No 2015/1375 of 10 August 2015 [46]. The farms followed the “all in–all out” principle. The animals had no access to a natural environment. The construction of the buildings prevented rodents, other mammals and birds from entering: rodents were prevented from digging into the area by a concrete area surrounding the building, the windows were carefully secured with nets and the lawns were neat and trimmed. In addition, no unnecessary objects were stored in the vicinity of the buildings, the waste bins were carefully closed and their design protected them from rodents; the site also implemented a properly-documented pest control program for both rodents and insects. The animal feed was produced in accordance with Regulation No. 183/2005 of 12 January 2005 [49]. The feed was stored in tightly-closed silos that were protected from rodents, and was fed to the animals automatically, directly from the silos. Each visit was recorded. Neither outsiders nor companion animals had access to the livestock buildings.

Finally, the study also included nine organic farms, which were located in the Kujawsko-Pomorskie voivodeship. The organic farms met the conditions of Council Regulation (EC) No. 834/2007 of 28 June 2007 [47] and Act of 25 June 2009 on organic farming [48]. The farming procedures were based on the following principles: selection of appropriate breeds, which were easily adaptable to the local conditions (viz*.* złotnicka pstra and puławska breeds); reproduction used natural mating only; the animals were born on organic farms; farming respected high animal welfare standards and met species-specific behavioral needs; pigs had access to the environment in open-air areas; feed originated from organic production and met the animal nutritional requirements, did not contain GMOs, and was not produced using GMOs. Animal-health management was based on disease prevention, and veterinary treatment was used where appropriate and under strict conditions.

More detailed data on the location of the tested farms and the numbers of sampled animals is given in Table 1.

**Table 1:** Data on location of farms and number of sampled animals

Group	Voivodeship	Farm number	Number of tested animals
Finishing pigs on farms with high hygiene standards	Lubelskie	1	33
		2	35
		3	99
Finishing pigs on farms with low hygiene standards	Mazowieckie	1	4
		2	1
		3	17
		4	3
		5	5
		6	2
		7	9
		8	6
		9	6
		10	8
		11	21
		12	10
		13	23
Finishing pigs on farm with high level of biosecurity	Wielkopolskie	1	40
Finishing pigs on organic farms	Kujawsko–Pomorskie	1	11
		2	20
		3	5
		4	8
		5	13
		6	29
		7	11
		8	8
		9	11
Pigs at < 3 months	Mazowieckie	1	20
	Zachodniopomorskie	2	20
	Wielkopolskie	3	23
Finishing pigs at 3–4 months	Mazowieckie	1	10
	Wielkopolskie	2	20
		3	30
Finishing pigs at 5–6 months	Wielkopolskie	1	10
		2	10
		3	10
		4	27
Sows at ≥ 9 months	Wielkopolskie	1	20
		2	4
		3	8
		4	6
		5	3
		6	7
		7	7
		8	8
		9	2
		10	41
		11	1
		12	1
		13	1
		14	1
		15	1
		16	1
		17	30

### Serum samples

For the analysis of housing system, serum samples were obtained from 438 finishing pigs aged 6 months. Of these, 167 were kept on the farms with high hygiene standards, 115 on the farms with low hygiene standards, 40 on the farm with a high level of biosecurity and 116 on the organic farms. These pigs were sampled randomly during slaughter (i.e. every third or fifth animal); however, all pigs from the organic farms were sampled.

Regarding the analysis of age, serum samples were obtained from 322 pigs of different ages from farms with high hygiene standards: of these 63 were aged under 3 months, 60 aged 3–4 months and 57 aged 5–6 months (final fattening stage), as well as 142 sows aged 9 months and older. The pigs aged under 3 months were kept on three farms located in the Mazowieckie, Zachodniopomorskie and Wielkopolskie voivodeships. Those aged 3–4 months originated from three farms: two located in the Wielkopolskie voivodeship and one located in the Mazowieckie voivodeship. The pigs aged 5–6 months originated from four farms, located in the Wielkopolskie voivodeship. Finally, the sows aged 9 months and older originated from 17 farms, located in the Wielkopolskie voivodeship. For this analysis, the pigs were sampled by field veterinarians during routine veterinary monitoring of infectious diseases. Selection was random by the veterinarian, and did not depend on animal health or visual signs.

In total, for both analyses, serum samples were obtained from 760 pigs.

### Enzyme linked immunosorbent assay analysis

The serum samples were tested with the PrioCHECK^®^ Toxoplasma Ab porcine ELISA test (Prionics, Switzerland) for detecting anti-*T. gondii* IgG antibodies in porcine serum and meat juice with an estimated sensitivity of 98.9% and specificity of 92.7% [50]. The test was performed according to the manufacturer's recommendations. The optical density (OD) was measured with an ELISA plate reader (Epoch, BioTek, USA) at 450 nm (reference filter 620 nm). The result for each sample was calculated using the following formula:

$$PP = \frac{{\left( {OD450\,{\text{nm}}\, Sample - OD450\,{\text{nm}} \,NC} \right)}}{{\left( {OD450\,{\text{nm}} \,PC - OD450\,{\text{nm}} \,NC} \right)}} \times 100$$

where PP is percentage of positivity, NC is the negative control and PC is the positive control.

The result was considered reliable if the control sera met the following criteria: mean OD_450_ PC > 1.2; mean PP of low positive control > 35%; mean OD_450_ NC < 0.150. Results with PP ≥ 20% were regarded as positive.

For sera giving positive results at a dilution of 1:50, two-fold dilutions were made. The sera were tested in dilutions of 1:100, 1:200, 1:400, 1:800, and 1:1600. The following antibody titers were determined: < 50, 50–99, 100–199, 200–399, 400–799, 800–1599, and > 1600.

### Statistical analysis

The seroprevalence of *T. gondii* in pigs was analysed with regard to (a) breeding conditions, i.e. comparing pigs from different housing systems or hygiene standards, and (b) age. In both cases, a generalized linear mixed model was used with binomial distribution and logit binding function.

Two separate models were created. In both models, the dependent variable was the presence of antibodies in the serum sample; however, in model (a), the independent variable was the breeding conditions, while in model (b) it was the age of the examined animals (< 3 months old; 3–4 months old; 5–6 months old; ≥ 9 months old). The analysed pigs came from 53 farms. The mean number of pigs tested per farm was 14. Farm ID number was fitted as a random effect in both models to account for repeated sampling of individual farms. The farm-type analysis (model a) was performed only for the slaughter pigs cohort; however, the age analysis (model b) was performed only with the on-farm cohort of the animals from the farm with high hygiene standards—this allowed the influence of breeding method to be to eliminated.

In both models, comparisons between groups were performed using the Bonferroni adjustment. Statistical analyses were performed in SPSS software (version 24.0, IBM Corporation, Armonk, NY, USA).

## Results

Antibodies to *T. gondii* were found in 193 of 760 (25.4%) tested animals. The highest prevalence was found in pigs from organic farms (61 out of 116 animals examined; 52.6%), followed by those on farms with low hygiene (47 of 115; 40.9%) and the farm with high hygiene standards (9 of 167; 5.4%). Anti-*T. gondii* antibodies were not found in any of the 40 tested pigs on the farm with the high level of biosecurity.

The age group analysis found the greatest prevalence in sows aged 9 months and older (62 out of 142 examined animals; 43.7%), with lower levels observed in pigs aged 5–6 months (7 of 57; 12.3%) and those aged under 3 months (7 of 63; 11.1%). None of the 60 tested pigs aged 3–4 months had specific antibodies. Detailed information on the presence of antibodies and antibody titers in pigs of different groups is shown in Table 2.

**Table 2:** Presence of anti-*Toxoplasma gondii* antibodies in analyzed porcine serum samples

Group	Number of tested animals	Number of seropositive animals (dilution 1:50; titer < 50)	Ranges of antibody titers in seropositive animals				
			50–99	100–199	200–399	400–799	800–1599
Finishing pigs on farms with high hygiene standards	167	9 (5.4%)	8 (4.8%)	0 (0%)	1 (0.6%)	0 (0%)	0 (0%)
Finishing pigs on farms with low hygiene standards	115	47 (40.9%)	17 (14.8%)	4 (3.5%)	1 (0.9%)	7 (6.1%)	5 (4.3%)
Finishing pigs on farm with high level of biosecurity	40	0 (0%)	0 (0%)	0 (0%)	0 (0%)	0 (0%)	0 (0%)
Finishing pigs on organic farms	116	61 (52.6%)	4 (3.45%)	2 (1.75%)	10 (8.6%)	16 (13.8%)	13 (11.2%)
Subtotal:	438	117 (26.7%)	29 (6.6%)	6 (1.4%)	12 (2.7%)	23 (5.3%)	18 (4.1%)
Pigs at < 3 months	63	7 (11.1%)	2 (3.15%)	2 (3.15%)	3 (4.8%)	0 (0%)	0 (0%)
Finishing pigs at 3–4 months	60	0 (0%)	0 (0%)	0 (0%)	0 (0%)	0 (0%)	0 (0%)
Finishing pigs at 5–6 months	57	7 (12.3%)	5 (8.8%)	0 (0%)	1 (1.75%)	0 (0%)	0 (0%)
Sows at ≥ 9 months	142	62 (43.7%)	6 (4.2%)	3 (2.1%)	17 (12%)	9 (6.3%)	0 (0%)
Subtotal:	322	76 (23.6%)	13 (4%)	5 (1.6%)	21 (6.5%)	9 (2.8%)	0 (0%)
Total:	760	193 (25.4%)	42 (5.5%)	11 (1.4%)	33 (4.3%)	32 (4.2%)	18 (2.4%)

**Fig. 1:**
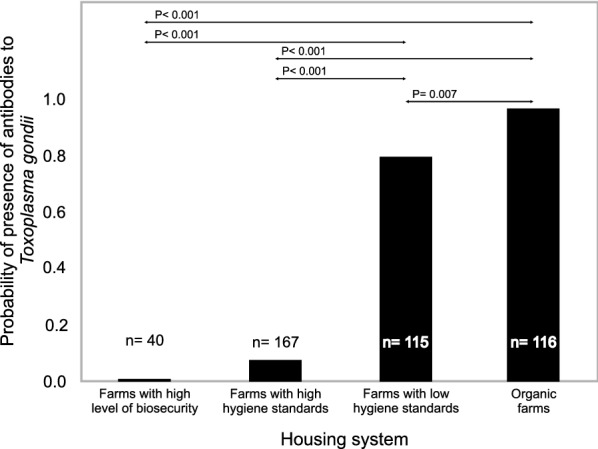
Mean frequency of antibodies to *Toxoplasma gondii* and pairwise comparison with Bonferroni correction (housing systems)

The generalized linear mixed binary model indicated significant differences in the degree of variation in antibody level between pigs from different housing systems and those from farms with different hygiene standards (F = 17.23; df = 3; P < 0.001), as well as those of different age groups (χ^2^ = 8.18; df = 3; P < 0.001). No difference was found between the high biosecurity farms and those with high hygiene standards (P = 0.184; Fig. 1); however, both groups demonstrated a significantly lower frequency of antibodies than the farm with low hygiene standards and the organic farms.

**Fig. 2:**
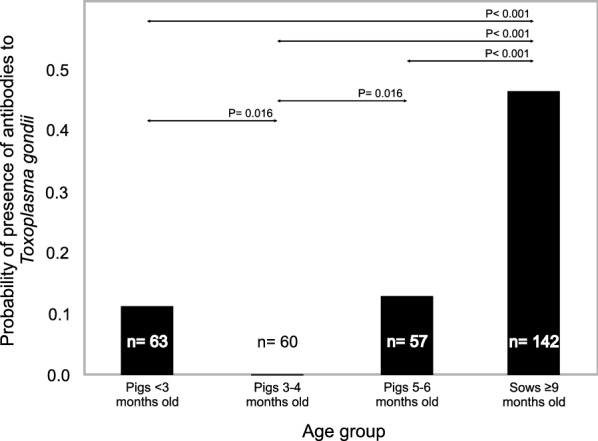
Mean frequency of antibodies to *Toxoplasma gondii* and pairwise comparison with Bonferroni correction (age groups)

Regarding age, the highest prevalence was observed in the sows aged 9 months and older. This value was significantly higher than in the groups aged under 3 months and those aged between 5 and 6 months; no significant difference was found between these two (P = 0.792; Fig. 2). Finally, both groups showed a significantly higher prevalence of antibodies than those aged 3–4 months.

## Discussion

In Europe, the percentage of *T. gondii* seropositivity has been found to range from 0 to 64% among pigs for slaughter and from 3 to 31% in sows [26]. However, no data exists on the prevalence of anti-*T. gondii* antibodies in pigs in Poland with regard to housing systems and the level of hygiene standards on farms. In addition, no research has been conducted on the prevalence of anti-*T. gondii* antibodies in pigs kept on organic farms in Poland.

While ELISA has been proposed as a routine screening test [10], the method may give false positive results and false negative results, the former as a result of cross reactivity from preexisting antibodies; this has been documented for a closely-related species, *Sarcocystis miescheriana* [51]. In both cases, i.e., false positives and false negatives, this has been attributed to the presence of interfering substances in the sample [51].

Experimentally-infected sows have been found to produce IgG class antibodies within two to three weeks of infection, and these antibodies remained for at least 38 weeks [52]. In pigs, the presence of anti-*T. gondii* antibodies correlates with the presence of parasite tissue cysts in the muscle tissue [11, 12, 28, 30]; this has been confirmed by comparing seroprevalence with a bioassay confirming or excluding the presence of infective tissue cysts [36]. Elsewhere, Hill et al. [53] found ELISA analysis of pig serum samples to demonstrate a 100% match with cat bioassay results; they also found MAT (Modified Agglutination Test) on the serum samples to demonstrate 80.64% match and ELISA on meat juice samples to demonstrate a 76.9% match. Hence, serological methods can play a valuable role in the diagnosis of *T. gondii* infections in slaughtered pigs.

In the present study, the fact that the highest proportion of seropositive animals were found on organic farms may be related to exposure of the pigs to the natural environment, which is known to increase the probability of *T. gondii* infection [10, 54]. This risk is associated with a higher probability of ingesting a rodent or coming into contact with feed, water or soil contaminated with oocysts [39, 55]. Moreover, rodents may be present in hay and silage [26]. The high percentage of seropositive animals identified in farms with low hygiene standards may be a consequence of inadequate housing protection from cats and rodents, which may lead to ingestion of oocysts excreted by cats or ingestion of intermediate hosts of *T. gondii*.

The farms with low hygiene standards were characterized by high proportion of seropositive animals. This may be related to the fact that they were small farms with a small number of animals; such systems are often associated with particular management practices which can increase the probability of infection, such as the use of a continuous cycle and of open food storage [10].

Farms where rodent control programs had been carefully implemented demonstrated lower numbers of seropositive pigs. Active rodent control programs have been found to have a positive impact on reducing the number of pigs presenting specific antibodies; the percentage of seropositive pigs dropped from 8–17% to 0–10% in three organic farms where rodent control was implemented for 4 months [56]. The prevalence of antibodies to *T. gondii* in pigs is also influenced by the type of rodent control method [57]: farms implementing programs using traps, bait and poison have fewer seropositive animals than those that use cats or dogs to control rodents [45], which is sometimes the case on organic farms [58]. In fact, the presence of cats on the farm is an important factor that increases the probability of infection among pigs [27, 59]: in previous studies, pigs kept on farms with a mean number of 6.8 or more cats were found to be more likely to demonstrate antibodies to *T. gondii* than those on farms with a mean number not exceeding 4.6 [2]. In addition, Feitosa et al. [60] report the presence of specific antibodies in 26.1% (23/88) of pigs on farms with cats, and 13.7% (14/102) on those without. It is important to note that farms can also be visited by feral cats [60, 61], and the lack of cats on the farm, or their small number, does not guarantee that the soil is not contaminated with *T. gondii* oocysts [62]. Interestingly, Limon et al. found the presence of cats on a farm, or the possibility for them to access the farm from outside, to not be significantly associated with *T. gondii* infection; however, a significant (2.6 fold) increase in infection risk was observed when cats had direct access to pig feed [10].

A higher chance of seropositivity was observed in pigs on organic farms compared to those in the high biosecurity level and high hygiene standards farms. This may be associated with a higher probability of ingestion of sporulated oocysts resulting from contact with contaminated soil e.g., during mud bathing or rooting [61]; indeed, the ingestion of ten sporulated oocysts is sufficient to infect a pig [26].

Complete elimination of the parasite from the environment seems impossible [63]. *T. gondii* DNA has been found in 17.8% (18/101) of tested soil samples [64] and in 9.7% (21/216) of samples of fruits and vegetables taken from shops and home gardens in Poland [65]. In addition, *T. gondii* DNA was recorded in 71 out of 243 (29.2%) soil samples from different sites in France; interestingly, the percentage of positive results fell from 38.3 to 19.7% as the distance from farms with cats increased [66]. Muñoz-Zanzi et al. [67] found oocyst-contaminated soil to be a source of infection among pigs; anti-*T. gondii* antibodies were identified in 8.8% (30/340) of pigs, of which 80% (24/30) had antibodies against the sporozoite-specific protein (TgERP).

Hill et al. [68] found pigs not housed in total confinement to be 7.7-times more likely to be infected with *T. gondii*. Also, van der Giessen et al. [3] claimed that free-range pigs are almost 16-times more likely to present specific antibodies than animals from intensive farming, and twice as likely as pigs from organic farms. Our present findings found the prevalence of anti-*T. gondii* antibodies to be 12-times higher in farms with low hygiene standards and 19-times higher in organic farms compared to farms with high hygiene standards. In addition, no specific antibodies were recorded in pigs on the farm with a high level of biosecurity, and only a low percentage was noted on farms with high hygiene standards; this was most likely related to the farm management system and most importantly, the high level of isolation and hygiene.

Seven of the 63 tested pigs aged under 3 months (11.1%) demonstrated antibodies to *T. gondii*. However, in pigs of this age, maternal antibodies may still be present [69] as they have been found for up to 4 months under experimental conditions [70]. In contrast, none of the pigs aged 3–4 months demonstrated antibodies (0%; 0/60); this may be attributed to the gradual decrease in the levels of maternal antibodies and the fact that they are unable to produce their own. The persistence of maternal antibodies in naturally-infected pigs may differ from that observed in experimentally-infected animals, and depends mainly on maternal antibody level: maternal antibodies persist longer in piglets from sows with higher antibody titers (≥ 1:500) than from those with low titers (≤ 1:50) [71]. Therefore, there is a need for special attention when assessing the prevalence of anti-*T. gondii* antibodies in pigs up to 4 months old, and routine testing of pigs at this age is unjustified due to the possible presence of maternal antibodies in the blood. Finally, pigs aged 5–6 months demonstrated a similar prevalence of antibodies as those younger than 3 months; however, this prevalence was lower than observed in the sows aged 9 months and older (43.7%; 62/142).

In the present study, sows aged 9 months were 10 times more likely to demonstrate antibodies to *T. gondii* than pigs aged 5–6 months. These findings indicate a higher prevalence of *T. gondii*-infected sows than in other European countries, where the percentage was found to range from 3 to 31% [26]. For example, 9.3% of the tested sows in Münsterland, Germany were seropositive [72] and 17.3% in Sweden [73]; in Serbia, which for years has had the highest number of seropositive animals, these values were 40.9% in 2006 and 30% in 2011 [42, 74].

Sows, pigs from farms with low hygiene standards, and pigs from organic farms, were more likely to have high antibody titers (≥ 1600) than those in other groups; this may indicate the presence of an acute infection [74, 75]. Animals with high antibody titers are more likely to present living parasites (tachyzoites) in the blood on analysis, thus indicating the presence of parasitemia. Its duration in pigs has not been determined [42]. Low titers may indicate chronic infection [75].

In Poland, consumption of commercially-produced and home-made sausages and cold meats is widespread. There is a high probability that any infection present in sows may be spread through the production of meat products, cold meat and sausages [9], as the production processes do not always inactivate the parasite. Live parasites have been isolated from raw sausages [76], raw ham [77] and Serrano ham [78], and a case of acute toxoplasmosis has been recorded after consumption of raw pork sausage [79].

Studies indicate a greater probability of isolating invasive tissue cysts at higher antibody titers [80, 81]. For example, Wang et al. [81] isolated invasive tissue cysts from 23 heart samples from 233 seropositive pigs: one seropositive at a serum dilution of 1:100, eight at 1:400, 13 at 1:800, and one at 1:1600. Specific antibodies were present in 22.2% of the tested animals. Invasive tissue cysts were found in 170 heart samples. Invasive tissue cysts were more frequently isolated from animals with higher antibody titers: 3.7% of sows with an antibody titer < 20, 37.1% with a titer of 20, 38.1% with a titer of 40, 60% with a titer of 80, 75% with a titer of 200, 77% with a titer of 400, 83% with a titer of 800 and 75.8% with a titer ≥ 2000 [82].

## Conclusions

Antibodies to *T. gondii* were most often found in pigs from organic and low-hygiene level farms, and in pigs aged 9 months and older. Meat derived from seropositive animals can pose a potential source of infection for humans. As maternal antibodies may be present in the blood of piglets up to 3–4 months of age, serological testing for *T. gondii* is unjustified in piglets up to 4 months of age.

## Data Availability

The datasets used and analysed during the current study are available from the corresponding author on reasonable request.

## References

[1] Fruh B, Bochicchio D, Edwards S, Hegelund L, Leeb C, Sundrum A (2014). Description of organic pig production in Europe. Org Agric.

[2] Kijlstra A, Meerburg BG, Bos AP (2009). Food safety in free-range and organic livestock systems: risk management and responsibility. J Food Prot.

[3] van der Giessen J, Fonville M, Bouwknegt M, Langelaar M, Vollema A (2007). Seroprevalence of *Trichinella spiralis* and *Toxoplasma gondii* in pigs from different housing systems in The Netherlands. Vet Parasitol.

[4] Karczewski G, Gołąb E (2011). Problemy diagnostyki toksoplazmozy wrodzonej. Przegląd epidemiologiczny.

[5] Zhang Y, Gong H, Mi R, Huang Y, Han X, Xia L (2020). Seroprevalence of *Toxoplasma gondii* infection in slaughter pigs in Shanghai. China. Parasitol Int.

[6] Račka K, Bártová E, Budíková M, Vodrážka P (2015). Survey of *Toxoplasma gondii* antibodies in meat juice of wild boar (*Sus scrofa*) in several districts of the Czech Republic. Ann Agric Environ Med.

[7] Olsen A, Berg R, Tagel M, Must K, Deksne G, Enemark HL (2019). Seroprevalence of *Toxoplasma gondii* in domestic pigs, sheep, cattle, wild boars, and moose in the Nordic-Baltic region: A systematic review and meta-analysis. Parasite Epidemiol Control..

[8] Hamilton CM, Kelly PJ, Bartley PM, Burrells A, Porco A, Metzler D (2015). *Toxoplasma gondii* in livestock in St. Kitts and Nevis. West Indies Parasit Vectors.

[9] Tenter AM, Heckeroth AR, Weiss LM (2000). *Toxoplasma gondii*: from animals to humans. Int J Parasitol.

[10] Limon G, Beauvais W, Dadios N, Villena I, Cockle C, Blaga R (2017). Cross-sectional study of *Toxoplasma gondii* infection in pig farms in England. Foodborne Pathog Dis.

[11] Deng H, Devleesschauwer B, Liu M, Li J, Wu Y, van der Giessen JWB (2018). Seroprevalence of *Toxoplasma gondii* in pregnant women and livestock in the mainland of China: a systematic review and hierarchical meta-analysis. Sci Rep.

[12] Sroka J, Bilska-Zając E, Wójcik-Fatla A, Zając V, Dutkiewicz J, Karamon J (2019). Detection and molecular characteristics of *Toxoplasma gondii* DNA in retail raw meat products in Poland. Foodborne Pathog Dis.

[13] Dubey JP, Cerqueira-Cézar CK, Murata FHA, Kwok OCH, Hill D, Yang Y (2020). All about *Toxoplasma gondii* infections in pigs: 2009–2020. Vet Parasitol.

[14] Milewska-Bobula B, Lipka B, Gołąb E, Dębski R, Marczyńska M, Paul M (2015). Recommended management of *Toxoplasma gondii* infection in pregnant women and their children. Przegl Epidemiol.

[15] Zhang XX, Ren WX, Tan QD, Hou G, Fei YC, Zhao LJ (2019). Meta-analysis of *Toxoplasma gondii* in pigs intended for human consumption in Mainland China. Acta Trop.

[16] Rostami A, Keshavarz H, Shojaee S, Mohebali M, Meamar AR (2014). Frequency of *Toxoplasma gondii* in HIV positive patients from west of Iran by ELISA and PCR. Iran J Parasitol.

[17] Pereira-Chioccola VL, Vidal JE, Su C (2009). *Toxoplasma gondii* infection and cerebral toxoplasmosis in HIV-infected patients. Future Microbiol.

[18] Montoya JG, Liesenfeld O (2004). Toxoplasmosis. Lancet.

[19] Shiadeh MN, Rostami A, Pearce BD, Gholipourmalekabadi M, Newport DJ, Danesh M (2016). The correlation between *Toxoplasma gondii* infection and prenatal depression in pregnant women. Eur J Clin Microbiol Infect Dis.

[20] Shiadeh MN, Behboodi Moghadam Z, Adam I, Saber V, Bagheri M, Rostami A (2017). Human infectious diseases and risk of preeclampsia: an updated review of the literature. Infection.

[21] Rostami A, Riahi SM, Fakhri Y, Saber V, Hanifehpour H, Valizadeh S (2017). The global seroprevalence of *Toxoplasma gondii* among wild boars: a systematic review and meta-analysis. Vet Parasitol.

[22] Fallahi S, Rostami A, Birjandi M, Zebardast N, Kheirandish F, Spotin A (2017). Parkinson’s disease and *Toxoplasma gondii* infection: sero-molecular assess the possible link among patients. Acta Trop.

[23] Rostami A, Seyyedtabaei SJ, Aghamolaie S, Behniafar H, Lasjerdi Z, Abdolrasouli A (2016). Seroprevalence and risk factors associated with *Toxoplasma gondii* infection among rural communities in northern Iran. Rev Inst Med Trop Sao Paulo.

[24] Shiadeh MN, Niyyati M, Fallahi S, Rostami A (2016). Human parasitic protozoan infection to infertility: a systematic review. Parasitol Res.

[25] Sutterland AL, Fond G, Kuin A, Koeter MW, Lutter R, van Gool T (2015). Beyond the association *Toxoplasma gondii* in schizophrenia, bipolar disorder, and addiction: systematic review and meta-analysis. Acta Psychiatr Scand.

[26] EFSA (2007). Monitoring of Toxoplasma in humans, food and animals. Scientific opinion of the panel on biological hazards. EFSA.

[27] Foroutan M, Fakhri Y, Riahi SM, Ebrahimpour S, Namroodi S, Taghipour A (2019). The global seroprevalence of *Toxoplasma gondii* in pigs: a systematic review and meta-analysis. Vet Parasitol.

[28] Campero LM, Schott F, Gottstein B, Deplazes P, Sidler X, Basso W (2020). Detection of antibodies to *Toxoplasma gondii* in oral fluid from pigs. Int J Parasitol.

[29] EFSA 2011 (2011). Technical specifications on harmonised epidemiological indicators for public health hazards to be covered by meat inspection of swine. EFSA J.

[30] Sroka J, Karamon J, Wójcik-Fatla A, Piotrowska W, Dutkiewicz J, Bilska-Zając E (2020). *Toxoplasma gondii* infection in slaughtered pigs and cattle in Poland: seroprevalence, molecular detection and characterization of parasites in meat. Parasit Vectors.

[31] Paul M (1998). Potencjalne źrodła zarażenia *Toxoplasma gondii* w przypadkach badanych w krótkim czasie po zarażeniu. Przegl Epid.

[32] Statistical Yearbook of Agriculture 2020. Statistics Poland, Warsaw 2020. ISSN 2080-8798.

[33] Jones JL, Dargelas V, Roberts J, Press C, Remington JS, Montoya JG (2009). Risk factors for *Toxoplasma gondii* infection in the United States. Clin Infect Dis.

[34] Fredericks J, Hawkins-Cooper DS, Hill DE, Luchansky JB, Porto-Fett ACS, Shoyer BA (2020). Inactivation of *Toxoplasma gondii* bradyzoites after salt exposure during preparation of dry-cured hams. J Food Prot.

[35] Juránková J, Basso W, Neumayerová H, Baláž V, Jánová E, Sidler X (2014). Brain is the predilection site of *Toxoplasma gondii* in experimentally inoculated pigs as revealed by magnetic capture and real-time PCR. Food Microbiol.

[36] Kijlstra A, Jongert E (2008). Control of the risk of human toxoplasmosis transmitted by meat. Int J Parasitol.

[37] Rani S, Cerqueira-Cézar CK, Murata FHA, Sadler M, Kwok OCH, Pradhan AK (2019). *Toxoplasma gondii* tissue cyst formation and density of tissue cysts in shoulders of pigs 7 and 14 days after feeding infected mice tissues. Vet Parasitol.

[38] Aspinall TV, Marlee D, Hyde JE, Sims PF (2002). Prevalence of *Toxoplasma gondii* in commercial meat products as monitored by polymerase chain reaction—food for thought?. Int J Parasitol.

[39] Guo M, Dubey JP, Hill D, Buchanan RL, Gamble HR, Jones JL (2015). Prevalence and risk factors for *Toxoplasma gondii* infection in meat animals and meat products destined for human consumption. J Food Prot.

[40] Kim JH, Kang KI, Kang WC, Sohn HJ, Jean YH, Park BK (2009). Porcine abortion outbreak associated with *Toxoplasma gondii* in Jeju Island. Korea J Vet Sci.

[41] Kofoed KG, Vorslund-Kiær M, Nielsen HV, Alban L, Johansen MV (2017). Sero-prevalence of *Toxoplasma gondii* in Danish pigs. Vet Parasitol Reg Stud Reports.

[42] Klun I, Vujanić M, Yera H, Nikolić A, Ivović V, Bobić B (2011). *Toxoplasma gondii* infection in slaughter pigs in Serbia: seroprevalence and demonstration of parasites in blood. Vet Res.

[43] Strategia odbudowy i rozwoju produkcji trzody chlewnej w Polsce do roku 2030 mająca na celu poprawę funkcjonowania sektora produkcji wieprzowiny. Pod red. Tadeusza Blicharskiego i Anny Hammermeister. Warszawa 2013. Sfinansowano ze Środkow Funduszu Promocji Mięsa Wieprzowego. Warszawa: Polski Związek Hodowcow i Producentow Trzody Chlewnej POLSUS.

[44] Djokic V, Fablet C, Blaga R, Rose N, Perret C, Djurkovic-Djakovic O (2016). Factors associated with *Toxoplasma gondii* infection in confined farrow-to-finish pig herds in western France: an exploratory study in 60 herds. Parasit Vectors.

[45] Herrero L, Gracia MJ, Perez-Arquillue C, Lazaro R, Herrera M, Herrera A (2016). *Toxoplasma gondii*: pig seroprevalence, associated risk factors and viability in fresh pork meat. Vet Parasitol.

[46] Comission Implementing Regulation (EU) 2015/1375 of 10 August 2015 laying down specific rules on official controls for *Trichinella* in meat.

[47] Council Regulation (EC) No 834/2007 of 28 June 2007 on organic production and labelling of organic products and repealing Regulation (EEC) No 2092/91.

[48] Ustawa z dnia 25 czerwca 2009 r. o rolnictwie ekologicznym. Dz.U. 2009 Nr 116 poz. 975.

[49] Regulation (EC) No 183/2005 of the European Parliament and of the Council of 12 January 2005 laying down requirements for feed hygiene.

[50] Basso W, Hartnack S, Pardini L, Maksimov P, Koudela B, Venturini MC (2013). Assessment of diagnostic accuracy of a commercial ELISA for the detection of *Toxoplasma gondii* infection in pigs compared with IFAT, TgSAG1-ELISA and Western blot, using a Bayesian latent class approach. Int J Parasitol.

[51] Olsen A, Alban L, Denwood M, Houe H, Jensen TB, Nielsen HV (2021). A longitudinal study of *Toxoplasma gondii* seroconversion on four large Danish sow farms. Vet Parasitol.

[52] Basso W, Grimm F, Ruetten M, Djokic V, Blaga R, Sidler X (2017). Experimental *Toxoplasma gondii* infections in pigs: humoral immune response, estimation of specific IgG avidity and the challenges of reproducing vertical transmission in sows. Vet Parasitol.

[53] Hill DE, Chirukandoth S, Dubey JP, Lunney JK, Gamble HR (2006). Comparison of detection methods for *Toxoplasma gondii* in naturally and experimentally infected swine. Vet Parasitol.

[54] Pablos-Tanarro A, Ortega-Mora LM, Palomo A, Casasola F, Ferre I (2018). Seroprevalence of *Toxoplasma gondii* in Iberian pig sows. Parasitol Res.

[55] Dubey JP, Hill DE, Rozeboom DW, Rajendran C, Choudhary S, Ferreira LR (2012). High prevalence and genotypes of *Toxoplasma gondii* isolated from organic pigs in northern USA. Vet Parasitol.

[56] Kijlstra A, Meerburg BG, Cornelissen J, De Craeye S, Vereijken P, Jongert E (2008). The role of rodents and shrews in the transmission of *Toxoplasma gondii* to pigs. Vet Parasitol.

[57] Kijlstra A, Eissen OA, Cornelissen J, Munniksma K, Eijck I, Kortbeek T (2004). *Toxoplasma gondii* infection in animal-friendly pig production systems. Invest Ophthalmol Vis Sci.

[58] Slany M, Reslova N, Babak V, Lorencova A (2016). Molecular characterization of *Toxoplasma gondii* in pork meat from different production systems in the Czech Republic. Int J Food Microbiol.

[59] Magalhães FJR, Ribeiro-Andrade M, Souza FM, Lima Filho CDF, Biondo AW, Vidotto O (2017). Seroprevalence and spatial distribution of *Toxoplasma gondii* infection in cats, dogs, pigs and equines of the Fernando de Noronha Island. Brazil Parasitol Int.

[60] Feitosa TF, Vilela VL, de Melo LR, de Almeida Neto JL, Souto DV, de Morais DF (2014). *Toxoplasma gondii* and *Neospora caninum* in slaughtered pigs from Northeast. Brazil Vet Parasitol.

[61] Wallander C, Frossling J, Dorea FC, Uggla A, Vagsholm I, Lunden A (2016). Pasture is a risk factor for *Toxoplasma gondii* infection in fattening pigs. Vet Parasitol.

[62] Ortega-Pacheco A, Acosta-Viana KY, Guzman-Marin E, Uitzil-Alvarez B, Rodriguez-Buenfil JC, Jimenez-Coello M (2011). Infection dynamic of *Toxoplasma gondii* in two fattening pig farms exposed to high and low cat density in an endemic region. Vet Parasitol.

[63] Sroka J, Szymańska J (2012). Analysis of prevalence of *Toxoplasma gondii* infection in selected rural households in the Lublin Region. Bull Vet Inst Pulawy.

[64] Lass A, Pietkiewicz H, Modzelewska E, Dumètre A, Szostakowska B, Myjak P (2009). Detection of *Toxoplasma gondii* oocysts in environmental soil samples using molecular methods. Eur J Clin Microbiol Infect Dis.

[65] Lass A, Pietkiewicz H, Szostakowska B, Myjak P (2012). The first detection of *Toxoplasma gondii* DNA in environmental fruits and vegetables samples. Eur J Clin Microbiol Infect Dis.

[66] Gotteland C, Gilot-Fromont E, Aubert D, Poulle ML, Dupuis E, Darde ML (2014). Spatial distribution of *Toxoplasma gondii* oocysts in soil in a rural area: Influence of cats and land use. Vet Parasitol.

[67] Muñoz-Zanzi C, Tamayo R, Balboa J, Hill D (2012). Detection of oocyst-associated toxoplasmosis in swine from southern Chile. Zoonoses Public Health.

[68] Hill DE, Haley C, Wagner B, Gamble HR, Dubey JP (2010). Seroprevalence of and risk factors for *Toxoplasma gondii* in the US swine herd using sera collected during the National Animal Health Monitoring Survey (Swine 2006). Zoonoses Public Health.

[69] Villari S, Vesco G, Petersen E, Crispo A, Buffolano W (2009). Risk factors for toxoplasmosis in pigs bred in Sicily Southern Italy. Vet Parasitol.

[70] Dubey JP, Urban JF (1990). Diagnosis of transplacentally induced toxoplasmosis in pigs. Am J Vet Res.

[71] Garcia-Bocanegra I, Simon-Grife M, Sibila M, Dubey JP, Cabezon O, Martin G (2010). Duration of maternally derived antibodies in *Toxoplasma gondii* naturally infected piglets. Vet Parasitol.

[72] Damriyasa IM, Bauer C (2005). Seroprevalence of *Toxoplasma gondii* infection in sows in Munsterland Germany. Dtsch Tierarztl Wochenschr.

[73] Lunden A, Lind P, Engvall EO, Gustavsson K, Uggla A, Vagsholm I (2002). Serological survey of *Toxoplasma gondii* infection in pigs slaughtered in Sweden. Scand J Infect Dis.

[74] Klun I, Djurković-Djaković O, Katić-Radivojević S, Nikolić A (2006). Cross-sectional survey on *Toxoplasma gondii* infection in cattle, sheep and pigs in Serbia: seroprevalence and risk factors. Vet Parasitol.

[75] Oliveira GC, de Souza Almeida HM, Sartori RS, Rossi GAM, de Oliveira LG, Langoni H (2018). Prevalence of *Toxoplasma gondii* infections in swine of non-tecnified rearing farms of the northeastern region of the state of São Paulo, Brazil and associated risk factors. Parasite Epidemiol Control.

[76] Abdulmawjood A, Rosa S, Taubert A, Bauer C, Failing K, Zahner H (2014). Investigation of persistence of infectious *Toxoplasma gondii* in raw sausages using in-house developed and validated real time-PCR. Meat Sci.

[77] Herrero L, Gracia MJ, Perez-Arquillue C, Lazaro R, Herrera A, Bayarri S (2017). *Toxoplasma gondii* in raw and dry-cured ham: the influence of the curing process. Food Microbiol.

[78] Gomez-Samblas M, Vílchez S, Racero JC, Fuentes MV, Osuna A (2015). Quantification and viability assays of *Toxoplasma gondii* in commercial “Serrano” ham samples using magnetic capture real-time qPCR and bioassay techniques. Food Microbiol.

[79] Vitale M, Tumino G, Partanna S, La Chiusa S, Mancuso G, Giglia ML (2014). Impact of traditional practices on food safety: a case of acute toxoplasmosis related to the consumption of contaminated raw pork sausage in Italy. J Food Prot.

[80] Dubey JP (2009). Toxoplasmosis in pigs—the last 20 years. Vet Parasitol.

[81] Wang D, Liu Y, Jiang T, Zhang G, Yuan G, He J (2016). Seroprevalence and genotypes of *Toxoplasma gondii* isolated from pigs intended for human consumption in Liaoning province, northeastern China. Parasit Vectors.

[82] Dubey JP, Thulliez P, Powell EC (1995). *Toxoplasma gondii* in Iowa sows: comparison of antibody titers to isolation of *T. gondii* by bioassays in mice and cats. J Parasitol.

